# County-Level Atrazine Use and Gastroschisis

**DOI:** 10.1001/jamanetworkopen.2024.10056

**Published:** 2024-05-06

**Authors:** Sunaya R. Krishnapura, Elizabeth McNeer, William D. Dupont, Stephen W. Patrick

**Affiliations:** 1 Vanderbilt University School of Medicine, Nashville, Tennessee; 2 Vanderbilt Center for Child Health Policy, Vanderbilt University Medical Center, Nashville, Tennessee; 3 Department of Biostatistics, Vanderbilt University Medical Center, Nashville, Tennessee; 4 Department of Pediatrics, Vanderbilt University Medical Center, Nashville, Tennessee; 5 Department of Health Policy, Vanderbilt University Medical Center, Nashville, Tennessee

## Abstract

**Importance:**

The incidence of gastroschisis, a birth defect involving the herniation of the small bowel through the abdominal wall, has increased in the US since the 1960s. The pesticide atrazine is a hypothesized cause of gastroschisis; however, examination of the association between atrazine and gastroschisis has been limited.

**Objective:**

To evaluate national trends in gastroschisis incidence, maternal and infant characteristics associated with gastroschisis, and whether county-level atrazine use is associated with gastroschisis.

**Design, Setting, and Participants:**

This retrospective, repeated cross-sectional study examined birth certificate data of all live births in the US and data on atrazine use from the US Geological Survey from January 1, 2009, through December 31, 2019. The data analysis was performed between August 5, 2021, and May 26, 2023.

**Exposures:**

County-level atrazine use.

**Main Outcomes and Measures:**

The primary outcome was gastroschisis incidence. Covariates included maternal age, race and ethnicity, body mass index (measured by weight in kilograms divided by height in meters squared), parity, insurance type, *Chlamydia* infection during pregnancy, smoking, and rurality. Mixed-effects logistic regression models (year fixed effects and county random effects) were constructed using different county-level atrazine exposure variables (1-, 5-, and 10-year means).

**Results:**

Between 2009 and 2019, 39 282 566 live births were identified, with 10 527 infant diagnoses of gastroschisis. Infants with gastroschisis were more likely to have mothers who identified as non-Hispanic White (61% vs 54%; *P* < .001), had a lower body mass index (median [IQR], 23.4 [20.8-27.2] vs 25.4 [22.0-30.8]; *P* < .001), were more likely to be nulliparous (median [IQR], 0 [0-1] vs 1 [0-2]; *P* < .001), and were more commonly covered by Medicaid (63% vs 43%; *P* < .001). During the study period, the rate (per 1000 live births) of gastroschisis decreased from 0.31 (95% CI, 0.29-0.33) to 0.22 (95% CI, 0.21-0.24). The median (IQR) county-level atrazine use estimates were higher among infants with gastroschisis (1 year, 1389 [IQR, 198-10 162] vs 1023 [IQR, 167-6960] kg; 5 years, 1425 [IQR, 273-9895] vs 1057 [IQR, 199-6926] kg; 10 years, 1508 [IQR, 286-10 271] vs 1113 [IQR, 200-6650] kg; *P* < .001). In adjusted models, higher county levels of atrazine (each 100 000-kg increase) were associated with a higher incidence of gastroschisis (1 year: adjusted odds ratio [AOR], 1.12 [95% CI, 1.01-1.24]; 5 years: AOR, 1.15 [95% CI, 1.02-1.30]; 10 years: AOR, 1.21 [95% CI, 1.07-1.38]).

**Conclusions and Relevance:**

In this cross-sectional study, higher county levels of atrazine were associated with infant diagnoses of gastroschisis. While atrazine is the second-most used herbicide in the US, numerous countries around the world have banned it out of concern for adverse effects on human health. These findings suggest that exploring alternatives to atrazine in the US may be warranted.

## Key Points

**Question:**

What is the association of county-level atrazine use with infant diagnoses of gastroschisis?

**Findings:**

In this cross-sectional study of nearly 40 million US births between 2009 and 2019, White race, young age, lower body mass index and parity, cigarette smoking, rurality, and *Chlamydia* infection during pregnancy were associated with gastroschisis. Additionally, higher levels of atrazine use at the county level were associated with an increased incidence of gastroschisis.

**Meaning:**

These findings suggest that exploring alternatives to atrazine in the US may be warranted.

## Teaser

This cross-sectional study evaluates US trends in gastroschisis incidence and maternal-infant characteristics and county-level atrazine use associated with gastroschisis.

## Introduction

Gastroschisis is a birth defect involving the abdominal wall that results in herniation of the small bowel and, at times, other abdominal organs.^1,2^ The condition requires immediate surgical attention following birth, resulting in prolonged hospital stays in neonatal intensive care units. Although surgical advancements have dramatically improved outcomes for infants born with the congenital abnormality, gastroschisis may be associated with substantial morbidity, including short bowel syndrome and intestinal atresia.^1,2^ Data suggest that rates of gastroschisis have increased globally,^3,4,5^ increasing from 0.06 to 0.8 per 10 000 births in the 1960s^6^ to 4.9 per 10 000 births in 2014.^5^ These increased rates have predominantly been observed in young White mothers^3,7^; however, data on the national incidence of gastroschisis at present are sparse.

Despite this recorded rise in incidence of gastroschisis, substantial gaps remain in our knowledge about the cause of the defect. Compared with other congenital anomalies, gastroschisis may be the result of the joint interplay of genetic and nongenetic factors.^6^ Several theories hypothesize the embryologic origins of gastroschisis, but few have successfully connected known risk factors, such as young age (<25 years), smoking, low body mass index (BMI) (as measured by weight in kilograms divided by height in meters squared), and nulliparity, to the anatomic basis of the defect.^6^ One predominating theory proposes that these various risk factors may contribute to the pathophysiology of gastroschisis via an estrogen-linked hypothesis.^8^ In standard embryologic development, the right-side umbilical vein involutes, leaving the umbilical cord with 2 umbilical arteries and the remaining left-side umbilical vein.^8,9^ This area of involution provides a site that is susceptible to clot formation, which is precipitated by increased levels of estrogen.^8,9^ Thrombus formation disrupts cell signaling and, consequently, the development of the abdominal wall, leading to the external protrusion of abdominal contents.^8,9^ While the association between gastroschisis and the established risk factors may be explained by the estrogen-linked hypothesis, it does not account for the increased rates of gastroschisis observed in the US over time.

Environmental factors, such as pesticide exposure, have also been associated with gastroschisis.^10^ Atrazine is an herbicide that was first approved for use in 1958 and has been applied to fields growing corn, sugarcane, sorghum, etc.^11,12^ Several studies have shown that atrazine may affect the reproductive development of frogs and rats and induce birth defects in rats.^10,11,12,13^ It has been hypothesized that these outcomes may be due to atrazine’s role as an endocrine disruptor.^8,9,10,13^ Consequently, atrazine may contribute to the formation of gastroschisis by functioning as an estrogen endocrine disruptor.^8,9,10^ However, studies investigating the association of atrazine exposure with incidence of gastroschisis have been limited to single states^10,14,15^ and counties.^16,17^

To address these knowledge gaps, our objective was to investigate more recent national trends in gastroschisis incidence and evaluate maternal and infant characteristics associated with gastroschisis. To expand on existing research, we also examined the association between atrazine use and gastroschisis at the national level. While the reasons for the increase in rates of gastroschisis may be multifactorial, we hypothesized that county-level exposure to atrazine would be associated with the increased incidence of gastroschisis, potentially explaining the observed trends.

## Methods

### Cohort and Data

In this analysis of retrospective, repeated cross-sectional data, we included data for all US births from January 1, 2009, to December 31, 2019. Birth data from the National Vital Statistics System were obtained from the National Center for Health Statistics at the Centers for Disease Control and Prevention. Pesticide data were acquired from the US Geological Survey (USGS). A report published in 2013 by the USGS outlines the methods regarding the collection of these data and generation of pesticide use estimates.^18,19^ The Vanderbilt University Medical Center institutional review board deemed this study exempt from human participant review, and informed consent was not required. This study followed the Strengthening the Reporting of Observational Studies in Epidemiology (STROBE) reporting guideline for cross-sectional studies.

### Conceptual Model

The estrogen-linked hypothesis provides a framework for understanding how different risk factors may exert effects at the molecular level to disrupt abdominal wall development in utero, leading to the development of gastroschisis (eFigure 1 in Supplement 1).^8,9^ Young age, low BMI, and nulliparity have been linked to elevated levels of estrogen, especially during the first trimester.^3,8,9,10^ Cigarette smoking has also been implicated in greater risk of gastroschisis.^1,2,3,8,9^ Although smoking is not associated with estrogen dysregulation, it has been associated with negative vascular outcomes, which aligns with the estrogen-linked hypothesis’s anatomic explanation of gastroschisis.^8,9^ The literature also has suggested an association with the increased risk of estrogen-mediated clotting among White women.^8^ Finally, some study results have indicated that mothers who present with sexually transmitted infections, most often *Chlamydia*, or urinary tract infections around the beginning of the first trimester were more likely to deliver babies with gastroschisis.^20,21^ It has been hypothesized that rather than these infections being responsible for the development of gastroschisis, high estrogen levels, which underlie the pathophysiology of the defect, may predispose mothers to contract these infections.^22^

### Exposure of Interest

Our exposure of interest was atrazine use. The USGS used 2 different methods to estimate pesticide use when a crop reporting district did not report pesticide use.^18,19^ The EPest-low method (hereafter referred to as low) assumed zero use, and the EPest-high method (hereafter referred to as high) treated it as missing data and used the rates from nearby crop reporting districts to estimate the rate. We include both estimates from USGS in our analysis.

In addition, we considered 3 variations of the atrazine use variable to account for timing of the exposure prior to delivery. The first was the mean county-level atrazine use in the year before the birth, which we refer to as the 1-year average. Next, to account for the potential long-term exposure to atrazine at the county level, we also calculated means of county-level atrazine use for 5 and 10 years before the birth.

### Outcomes and Covariates

Incidence of gastroschisis, the outcome of interest, was measured using birth certificate data. Covariates included maternal age, maternal race and ethnicity (Hispanic; non-Hispanic Black; non-Hispanic White; and other [Alaska Native, Asian or Pacific Islander, non-Hispanic American Indian], given the low collective incidence of gastroschisis), BMI, number of previous births, birth payment source (Medicaid, private insurance, self-pay, other), *Chlamydia* infection during the pregnancy, smoking during the pregnancy, and rurality (urban, rural adjacent, rural remote). Information regarding covariates, including race and ethnicity, were obtained from birth certificate data from the National Vital Statistics System. As mentioned previously, research has identified differences in prevalence of gastroschisis among different racial and ethnic groups, with higher prevalence found in non-Hispanic White mothers.^3,7^

### Statistical Analysis

Analyses were conducted between August 5, 2021, and May 26, 2023. We compared characteristics of maternal-infant dyads with and without a diagnosis of gastroschisis using medians and IQRs for continuous variables and frequencies and percentages for categorical variables. We tested for differences between the groups using Wilcoxon rank sum tests and χ^2^ tests. Additionally, we created line plots to examine the rates of gastroschisis over time both overall and stratified by cigarette use, rurality, and payer.

We also evaluated the geographic distribution of gastroschisis and atrazine use by the 4 US census regions (Northeast, Midwest, South, West) and created maps of the county-level data. The χ^2^ and Kruskal-Wallis tests were used to test for differences in gastroschisis rates and mean atrazine use, respectively, among the census regions. Atrazine data were missing for 3.6% of county-years. In 1-year lagged models, these counties were not included; however, in the 5- and 10-year average model, the mean exposure over the time period was considered without including missing county-year data (ie, if 4 years of data were available within a 5-year period, the mean was calculated using the available 4 years).

Our mixed-effects models included fixed effects for years as an indicator variable and random effects for counties to account for correlation within county. Using these mixed-effects logistic regression models, we examined the association between county-level atrazine use and presence of gastroschisis while adjusting for rurality, maternal age, maternal race and ethnicity, maternal BMI, cigarette use, number of previous births, and *Chlamydia* infection during pregnancy, calendar year, county of residence, and payment source. In these models, all covariates were fixed effects except county of residence, which was a random effect. Separate models were constructed using each of the atrazine county-level exposure variables, ie, 1-year, 5-year, and 10-year averages. We used the high values for the primary analysis. All tests were 2-sided with a threshold of *P* < .05 for statistical significance. The models were run using Stata, version 15.1 (StataCorp LLC), and the maps were generated using the ggplot2 and urbnmapr packages of R, version 4.2.1 (R Foundation for Statistical Computing).

We conducted supplementary analyses to assess the robustness of our findings. In the first supplementary analysis, we fit the same regression models with the low values for the atrazine variables. In the other supplementary analyses, we excluded births in California because the atrazine data in this state were collected differently compared with the other states.^18,19^

## Results

Between 2009 and 2019, 39 282 566 live infants were born in the US, among whom 10 527 were diagnosed with gastroschisis. Infants with gastroschisis were more likely to have a lower birth weight (median [IQR], 2410 [2050-2780] g vs 3310 [2974-3629] g; *P* < .001) and earlier gestational age (median [IQR], 36 [35-37] vs 39 [38-40] weeks; *P* < .001). However, there was no association between gastroschisis and other obstetric complications, including prepregnancy and gestational diabetes, hypertensive disorders of pregnancy, and previous preterm birth (Table 1).

**Table 1.** Characteristics of Maternal-Infant Dyads With and Without a Diagnosis of Gastroschisis, US 2009-2019

Characteristic	Median (IQR) or No. of dyads (%)		*P* value
	With gastroschisis (n = 10 527)	Without gastroschisis (n = 39 272 039)	
Maternal age, y	22 (20-26)	28 (24-33)	<.001^a^
Maternal race and ethnicity			
Hispanic	2511 (24)	9 391 962 (24)	<.001^b^
Non-Hispanic Black	1140 (11)	5 812 656 (15)	
Non-Hispanic White	6382 (61)	21 096 006 (54)	
Other^c^	494 (5)	2 971 415 (8)	
Maternal BMI	23.4 (20.8-27.2)	25.4 (22.0-30.8)	<.001^a^
Smoking status^d^			
Yes	1914 (18)	2 934 636 (7)	<.001^b^
No	8079 (77)	34 610 514 (88)	
Previous births	0 (0-1)	1 (0-2)	<.001^a^
Birth weight, g	2410 (2050-2780)	3310 (2974-3629)	<.001^a^
Gestational age, wk	36 (35-37)	39 (38-40)	<.001^a^
Prepregnancy diabetes^d^			
Yes	52 (0)	320 317 (1)	<.001^b^
No	10 449 (99)	38 913 018 (99)	
Gestational diabetes^d^			
Yes	245 (2)	2 201 266 (6)	<.001^b^
No	10 256 (97)	37 032 069 (94)	
Prepregnancy hypertension^d^			
Yes	83 (1)	649 044 (2)	<.001^b^
No	10 418 (99)	38 584 291 (98)	
Gestational hypertension^d^			
Yes	305 (3)	2 189 988 (6)	<.001^b^
No	10 196 (97)	37 043 347 (94)	
Hypertension eclampsia^d^			
Yes	18 (0)	93 639 (0)	<.001^b^
No	10 483 (100)	39 139 696 (100)	
Previous preterm birth^d^			
Yes	337 (3)	1 108 224 (3)	<.001^b^
No	10 164 (97)	38 125 111 (97)	
*Chlamydia* infection during pregnancy^d^			
Yes	436 (4)	708 768 (2)	<.001^b^
No	10 040 (95)	38 464 564 (98)	
Rurality^e^			
Rural adjacent	1428 (14)	3 533 050 (9)	<.001^b^
Rural remote	841 (8)	1 923 456 (5)	
Urban	8258 (78)	33 815 345 (86)	
Payment			
Medicaid	6559 (63)	16 815 992 (43)	<.001^b^
Other^f^	491 (5)	1 698 427 (4)	
Private insurance	3075 (30)	18 720 960 (48)	
Self-pay	295 (3)	1 629 251 (4)	
WIC^d^			
Yes	5796 (55)	16 282 751 (41)	<.001^b^
No	4503 (43)	22 290 244 (57)	

Abbreviations: BMI, body mass index (as measured by weight in kilograms divided by height in meters squared); WIC, Special Supplemental Nutrition Program for Women, Infants, and Children.

^a^ By Wilcoxon rank sum test.

^b^ By χ^2^ test.

^c^ Includes Alaska Native, Asian or Pacific Islander, and non-Hispanic American Indian.

^d^ Percentages may not add up to 100 due to missingness.

^e^ Defined using Rural-Urban Continuum Codes (rural adjacent, 4, 6, or 8; rural remote, 5, 7, or 9; urban, 1, 2, or 3).

^f^ Includes Indian Health Service, TRICARE (formerly, Civilian Health and Medical Program of Uniformed Service), other government (federal, state, local), and other.

Among mothers who had babies with gastroschisis, 6382 (61%) identified as non-Hispanic White (hereafter White) compared with 2511 (24%) who identified as Hispanic, 1140 (11%) as non-Hispanic Black (hereafter Black), and 494 (5%) as other race and ethnicity. In contrast, among mothers who had babies without gastroschisis, 54% identified as White compared with 15% who identified as Black, 24% as Hispanic, and 8% as other race and ethnicity. Mothers of babies with gastroschisis were also more likely to be younger (median [IQR] age, 22 [20-26] vs 28 [24-33] years; *P* < .001); have a lower median BMI (median [IQR], 23.4 [20.8-27.2] vs 25.4 [22.0-30.8]; *P* < .001); have been nulliparous (median [IQR], 0 [0-1] vs 1 [0-2]; *P* < .001); live in more rural environments (22% vs 14%; *P* < .001); receive care covered by Medicaid (63% vs 43%; *P* < .001); and be enrolled in the Special Supplemental Nutrition Program for Women, Infants, and Children (55% vs 41%; *P* < .001). Maternal smoking (18% vs 7%; *P* < .001) and *Chlamydia* infection during pregnancy (4% vs 2%; *P* < .001) were also found to be associated with gastroschisis (Table 1).

**Figure 1.**
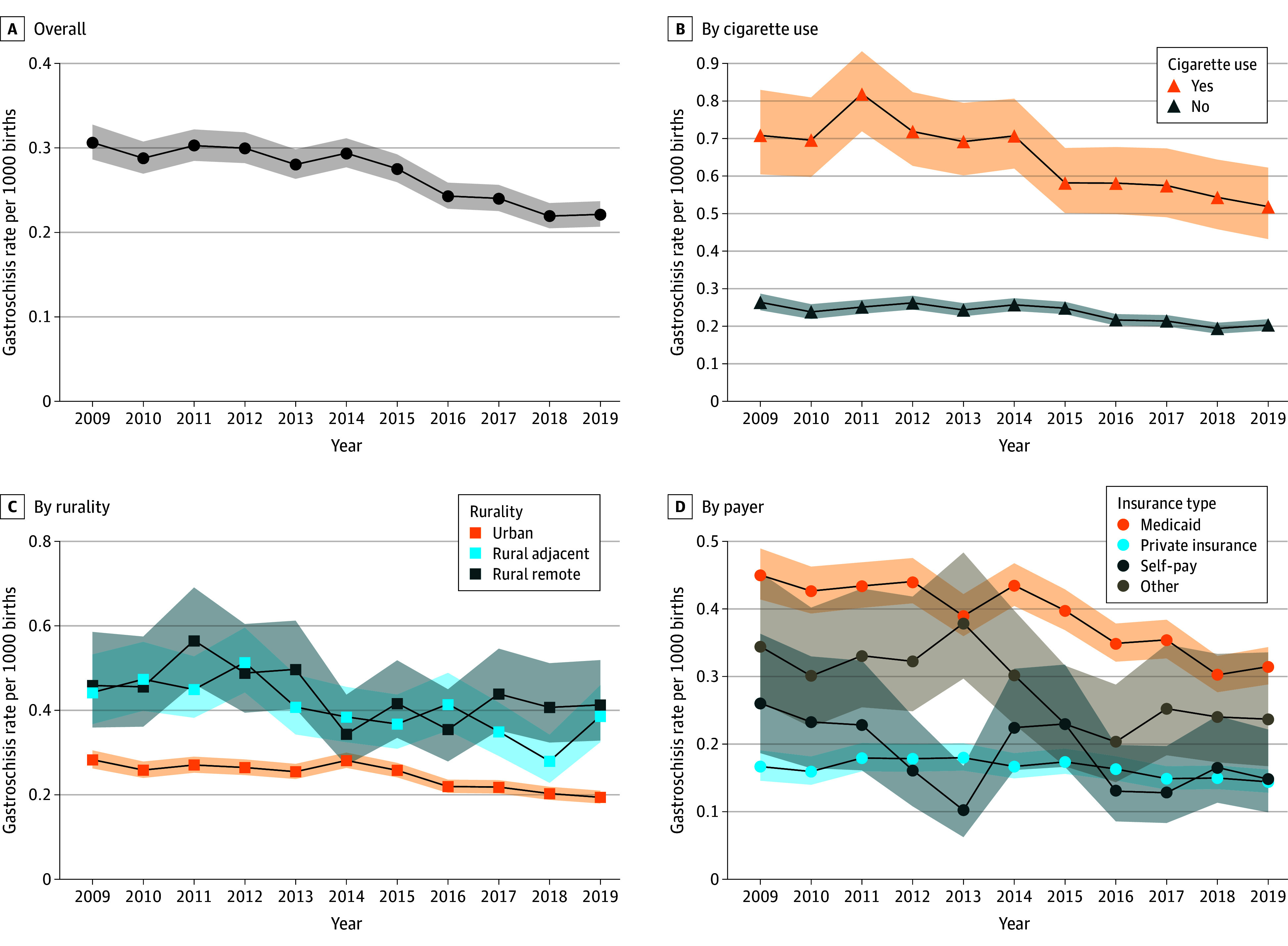
Rates of Gastroschisis in the US, 2009-2019 Shaded areas indicate 95% CIs.

During the study period, the rate of gastroschisis (per 1000 live births) in the US decreased from 0.31 (95% CI, 0.29-0.33) to 0.22 (95% CI, 0.21-0.24). Along with the fall in the national rate of gastroschisis from 2009 to 2019, the rate of gastroschisis (per 1000 live births) among mothers who smoked also decreased from 0.71 (95% CI, 0.60-0.82) to 0.52 (95% CI, 0.42-0.61). Similar declines were observed in mothers who lived in rural areas (remote rural, from 0.46 [95% CI, 0.35-0.57] to 0.41 [95% CI, 0.32-0.51] per 1000 live births; rural adjacent, from 0.44 [95% CI, 0.36-0.52] to 0.39 [95% CI, 0.32-0.45] per 1000 live births) compared with urban areas (from 0.28 [95% CI, 0.26-0.30] to 0.19 [95% CI, 0.18-0.21] per 1000 live births). Additionally, the rates among mothers insured by Medicaid decreased (from 0.45 [95% CI, 0.41-0.49] to 0.32 [0.29-0.34] per 1000 live births) to a greater extent compared with mothers with private insurance (from 0.17 [95% CI, 0.14-0.19] to 0.14 [0.13-0.16] per 1000 live births) (Figure 1).

### Association of County-Level Atrazine Use With Gastroschisis

**Figure 2.**
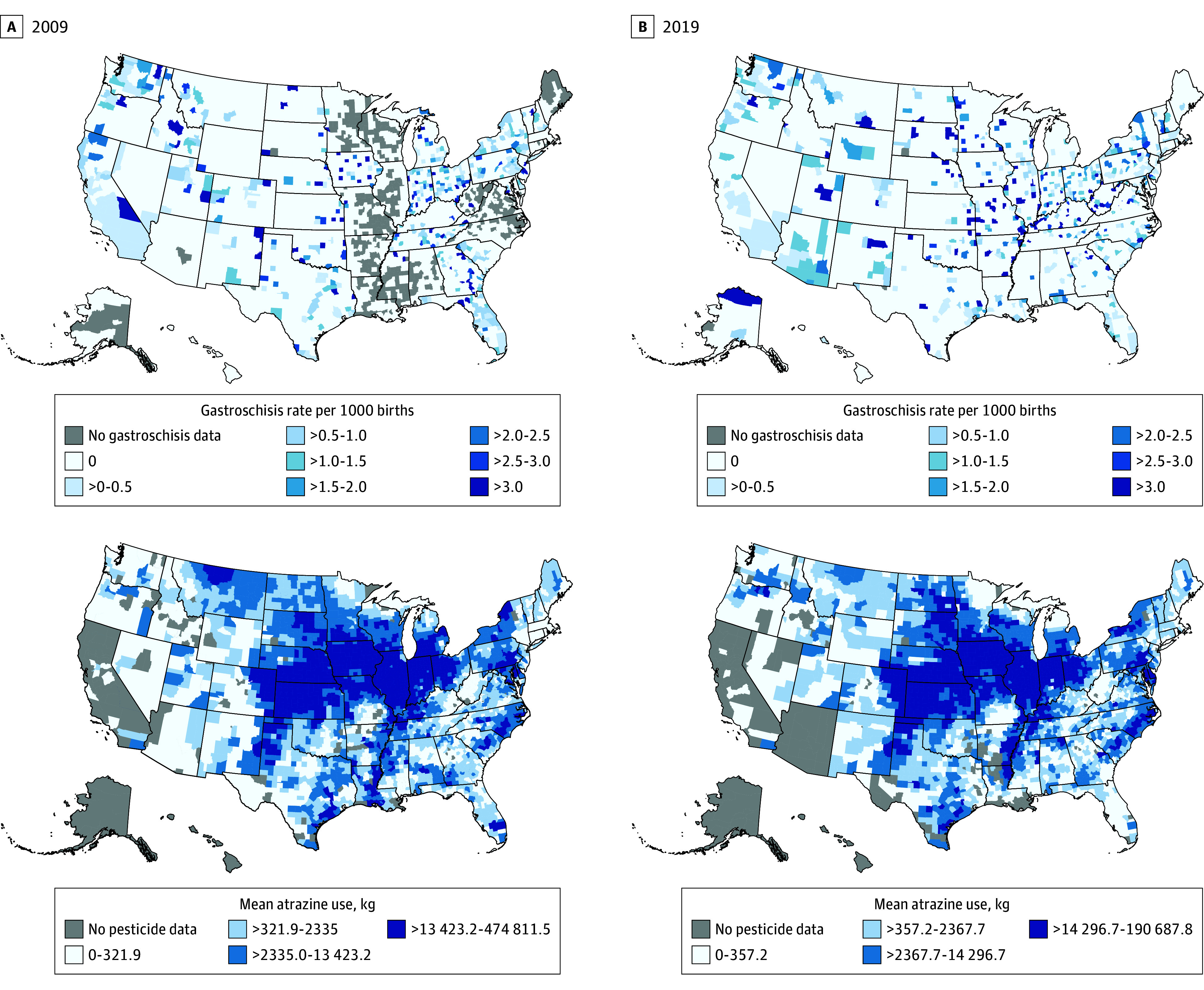
Gastroschisis Incidence and Atrazine Use

From 2009 to 2019, a higher proportion of infants with gastroschisis were born in the Midwest compared with other US Census regions (Figure 2; eTable 1 in Supplement 1). Notably, atrazine use from 2009 to 2019 has largely been concentrated in the Midwest, with a mean (SD) use of 22 702.1 (1399.1) kg of atrazine per year within this period. Some counties in the South and on the East Coast have also reported high use of atrazine. In 2009, the highest recorded use of atrazine in a county was 474 811.5 kg, while in 2019, the highest estimate was 190 687.8 kg (Figure 2). From 2009 to 2019, the mean (SD) county-level atrazine use increased from 10 343.6 (18 325.2) kg to 11 640.1 (19 958.7) kg (eFigure 2 in Supplement 1). In examining the association between county-level atrazine use and gastroschisis, all variations of the exposure variable showed that higher median estimates of atrazine use were associated with gastroschisis. Over the previous year, the median estimate of county-level atrazine use was 1389 kg (IQR, 198-10 162 kg) among infants with gastroschisis compared with 1023 kg (IQR, 167-6960 kg) among infants without the diagnosis. The corresponding medians over longer durations were 1425 kg (IQR, 273-9895 kg) vs 1057 kg (IQR, 199-6926 kg) averaged over 5 years and 1508 kg (IQR, 286-10 271 kg) vs 1113 kg (IQR, 200-6650 kg) averaged over 10 years (*P* < .001) (Table 2).

**Table 2.** Unadjusted Association of County-Level Atrazine Use With Gastroschisis Diagnosis, US 2009-2019

County-level atrazine use	Median (IQR), kg		*P* value
	Gastroschisis (n = 10 527)	No gastroschisis (n = 39 272 039)	
Previous year	1389 (198-10 162)	1023 (167-6960)	<.001
10-y Average	1508 (286-10 271)	1113 (200-6650)	<.001
5-y Average	1425 (273-9895)	1057 (199-6926)	<.001

### Multivariable Analysis

In multivariable analyses accounting for a priori–established covariates, year fixed effects, and county random effects, we found that higher atrazine use (each 100 000-kg increase) was associated with an increased risk of gastroschisis in all models of atrazine use (1 year: adjusted odds ratio [AOR], 1.12 [95% CI, 1.01-1.24]; 5 years: AOR, 1.15 [95% CI, 1.02-1.30]; 10 years: AOR, 1.21 [95% CI, 1.07-1.38]) (Table 3). Supplementary analyses yielded similar results (eTables 2-4; eFigures 3-5 in Supplement 1).

**Table 3.** Multivariable Analysis^a^ of Individual and County-Level Characteristics and Exposures Associated With Gastroschisis, US 2009-2019

Characteristic	Model 1: 5-y average of atrazine use (n = 32 152 331)		Model 2: 10-y average of atrazine use (n = 33 121 551)		Model 3: previous year atrazine use (n = 30 740 211)	
	AOR (95% CI)	*P* value	AOR (95% CI)	*P* value	AOR (95% CI)	*P* value
**County level**						
Atrazine use (100 000-kg increase)	1.15 (1.02-1.30)	.02	1.21 (1.07-1.38)	.003	1.12 (1.01-1.24)	.04
Rurality						
Urban	1 [Reference]	NA	1 [Reference]	NA	1 [Reference]	NA
Rural adjacent	1.07 (0.99-1.15)	.09	1.07 (0.99-1.15)	.077	1.07 (0.99-1.15)	.08
Rural remote	1.12 (1.02-1.22)	.02	1.12 (1.02-1.23)	.016	1.10 (1.00-1.21)	.04
**Individual level**						
Maternal age	0.87 (0.86-0.87)	<.001	0.87 (0.86-0.87)	<.001	0.87 (0.86-0.87)	<.001
Maternal race and ethnicity						
Hispanic	0.89 (0.83-0.94)	<.001	0.89 (0.84-0.95)	<.001	0.89 (0.84-0.95)	<.001
Non-Hispanic Black	0.56 (0.52-0.61)	<.001	0.56 (0.52-0.61)	<.001	0.56 (0.52-0.61)	<.001
Non-Hispanic White	1 [Reference]	NA	1 [Reference]	NA	1 [Reference]	NA
Other^b^	0.79 (0.71-0.88)	<.001	0.78 (0.70-0.87)	<.001	0.82 (0.73-0.91)	<.001
Maternal BMI	0.94 (0.94-0.94)	<.001	0.94 (0.94-0.94)	<.001	0.94 (0.94-0.94)	<.001
Cigarette use	1.69 (1.60-1.79)	<.001	1.70 (1.60-1.80)	<.001	1.69 (1.60-1.79)	<.001
No. of previous births	0.91 (0.89-0.94)	<.001	0.91 (0.89-0.94)	<.001	0.91 (0.89-0.93)	<.001
*Chlamydia* infection during pregnancy	1.15 (1.03-1.28)	.01	1.15 (1.03-1.28)	.01	1.14 (1.03-1.27)	.01
Payment						
Medicaid	1 [Reference]	NA	1 [Reference]	NA	1 [Reference]	NA
Private insurance	0.73 (0.69-0.77)	<.001	0.73 (0.69-0.77)	<.001	0.73 (0.69-0.77)	<.001
Self-pay	0.63 (0.55-0.72)	<.001	0.63 (0.56-0.72)	<.001	0.63 (0.55-0.72)	<.001
Other^c^	0.89 (0.80-0.98)	.02	0.89 (0.81-0.99)	.03	0.89 (0.81-0.99)	.03

Abbreviations: AOR, adjusted odds ratio; BMI, body mass index; NA, not applicable.

^a^ Models account for year fixed effects, county random effects, and use of high estimates of atrazine use.

^b^ Includes Asian or Pacific Islander, Alaska Native, and non-Hispanic American Indian.

^c^ Includes Indian Health Service, TRICARE (formerly, Civilian Health and Medical Program of Uniformed Service), other government (federal, state, local), and other.

## Discussion

In this national study of all US live births between 2009 and 2019, we found that county-level atrazine use was associated with infant diagnoses of gastroschisis. Additionally, we found that the incidence of gastroschisis has declined slightly in recent years. Prior estimates of the national incidence of gastroschisis are limited to post 2014. Like previous research,^1,2,3,8,9^ we also found that the diagnosis was more common among previously nulliparous women who identified as White, had a lower median BMI, lived in rural areas, smoked cigarettes, and reported a *Chlamydia* infection during pregnancy.

Our study found that atrazine use was highest in the midwestern region of the US, where rates of gastroschisis were similarly the highest compared with other census regions. Within our model of atrazine use averaged over a 10-year period, a 100 000-kg increase in average atrazine use in a mother’s county of residence was associated with a 21% increase in the odds of an infant being born with gastroschisis, after accounting for covariates and year fixed effects and county random effects. This association was similarly observed when atrazine use was averaged over 1-year and 5-year periods. Aside from our study, others conducted in Washington, Indiana, Hawaii, Texas, and California have also suggested that pesticides, in particular atrazine, may be associated with an increased prevalence of gastroschisis.^10,14,15,16,17,23^ One study investigating this correlation in Texas found that the probability of a birth complicated by gastroschisis as a result of atrazine exposure may vary by age, with the risk being greater in mothers 25 years or older.^14^ Future studies investigating the association between national atrazine use and gastroschisis may reveal differences in risk stratified by age.

The Centers for Disease Control and Prevention estimates that each year, more than 70 million pounds of atrazine are used in the US.^12^ Aside from gastroschisis, atrazine has also been associated with preterm birth and low birth weight.^24,25^ While our study did not assess biomarkers of atrazine exposure, other studies have shown an association between atrazine exposure and adverse birth outcomes using urinary biomarkers.^26^ The findings from the PELAGIE (Perturbateurs endocriniens: Étude Longitudinale sur les Anomalies de la Grossesse, l’Infertilité et l’Enfance) birth cohort in France showed an association between atrazine exposure and fetal growth restriction and small head circumference.^26^ Future research using urinary biomarkers may find a stronger association between atrazine exposure and gastroschisis incidence.

Although rates of gastroschisis have appeared to decline, the condition is still associated with considerable infant morbidity. Moreover, surgical repair of the defect has been associated with longer hospital stays and additional costs upward of $300 000, placing an enormous financial burden on families.^3^ Atrazine is the second-most used herbicide in the US, and widespread application of the pesticide has raised several concerns with regard to its effects on biodiversity.^11,12,27^ Numerous countries, including the European Union, have banned the substance out of concerns for its adverse effects on human health.^28^ Our study findings suggest that reexamination of policy regarding atrazine use may be warranted.

### Limitations

Our study has limitations that are common among studies of this type. First, birth certificate data may be subject to misclassification bias, with errors of omission or commission. Second, some variations in pesticide use data collection were present at the state level, and some data were missing for states at different time points. To address these limitations, we used 3 models and calculated means of county-level atrazine use for 5 and 10 years before birth to minimize the amount of missingness for our exposure of interest. We also made use of supplementary analyses excluding states with missing data to assess the robustness of our findings. Third, atrazine use data obtained from the USGS from 2018 and 2019 were listed as preliminary estimates. Final estimates are projected to be available in 2025. Fourth, aside from the covariates we identified, there may have been other confounding variables unaccounted for in this study, such as alcohol or opioid use.^8,9,29^ At present, the data on the association between opioid use and gastroschisis are limited; however, shared downstream pathways between the estrogen receptor and opioid receptor may explain how opioid use fits with the estrogen-linked hypothesis.^30^ Fifth, while our study made use of reported atrazine use data at the county level, we did not examine direct exposure of mothers to atrazine through the use of biomarkers. Finally, our results indicate an association between county-level atrazine use and incidence of gastroschisis but does not necessarily imply causation.

## Conclusions

In summary, this cross-sectional study found that higher county levels of atrazine were associated with infant diagnoses of gastroschisis. While atrazine is the second-most used herbicide in the US, numerous countries around the world have banned it out of concern for adverse effects on human health. These findings suggest that exploring alternatives to atrazine in the US may be warranted.

## Notes

Supplement 1. **eFigure 1**. Pathophysiology Model of Gastroschisis Based on the Estrogen-Linked Hypothesis **eTable 1.** Distribution of County-Level Atrazine Use and Gastroschisis by US Census Region **eFigure 2.** Overall Rate of Atrazine Use in the US, EPest-High and EPest-Low Methods **eTable 2.** Mixed-Effects Logistic Regression Model of Individual- and County-Level Characteristics and Exposures Associated With Gastroschisis, US 2009-2019, Including Births in California and Using EPest-Low **eFigure 3.** Mixed-Effects Logistic Regression Model of Individual- and County-Level Characteristics and Exposures Associated With Gastroschisis, US 2009-2019, Including Births in California and Using EPest-Low **eTable 3.** Mixed-Effects Logistic Regression Model of Individual- and County-Level Characteristics and Exposures Associated with Gastroschisis, US 2009-2019, Excluding Births in California and Using EPest-High **eFigure 4.** Mixed-Effects Logistic Regression Model of Individual- and County-Level Characteristics and Exposures Associated With Gastroschisis, US 2009-2019, Excluding Births in California and Using EPest-High **eTable 4.** Mixed-Effects Logistic Regression Model of Individual- and County-Level Characteristics and Exposures Associated With Gastroschisis, US 2009-2019, Excluding Births in California and Using EPest-Low **eFigure 5.** Mixed-Effects Logistic Regression Model of Individual- and County-Level Characteristics and Exposures Associated With Gastroschisis, US 2009-2019, Excluding Births in California and Using EPest-Low

Supplement 2.
Data Sharing Statement
