# Supplementary material for: County-Level Atrazine Use and Gastroschisis
Source: JAMA Netw Open. 2024 May 6;7(5):e2410056. doi: 10.1001/jamanetworkopen.2024.10056 (PMC11074809; doi:10.1001/jamanetworkopen.2024.10056)
Supplement: Supplement 1. — eFigure 1. Pathophysiology Model of Gastroschisis Based on the Estrogen-Linked Hypothesis eTable 1. Distribution of County-Level Atrazine Use and Gastroschisis by US Census Region eFigure 2. Overall Rate of Atrazine Use in the US, EPest-High and EPest-Low Methods eTable 2. Mixed-Effects Logistic Regression Model of Individual- and County-Level Characteristics and Exposures Associated With Gastroschisis, US 2009-2019, Including Births in California and Using EPest-Low eFigure 3. Mixed-Effects Logistic Regression Model of Individual- and County-Level Characteristics and Exposures Associated With Gastroschisis, US 2009-2019, Including Births in California and Using EPest-Low eTable 3. Mixed-Effects Logistic Regression Model of Individual- and County-Level Characteristics and Exposures Associated with Gastroschisis, US 2009-2019, Excluding Births in California and Using EPest-High eFigure 4. Mixed-Effects Logistic Regression Model of Individual- and County-Level Characteristics and Exposures Associated With Gastroschisis, US 2009-2019, Excluding Births in California and Using EPest-High eTable 4. Mixed-Effects Logistic Regression Model of Individual- and County-Level Characteristics and Exposures Associated With Gastroschisis, US 2009-2019, Excluding Births in California and Using EPest-Low eFigure 5. Mixed-Effects Logistic Regression Model of Individual- and County-Level Characteristics and Exposures Associated With Gastroschisis, US 2009-2019, Excluding Births in California and Using EPest-Low [file jamanetwopen-e2410056-s001.pdf]

Supplemental Online Content

Krishnapura SR, McNeer E, Dupont WD, Patrick SW. County-level atrazine use and gastroschisis. *JAMA Netw Open*. 2024;7(5):e2410056.
doi:10.1001/jamanetworkopen.2024.10056

eFigure 1. Pathophysiology Model of Gastroschisis Based on the Estrogen-Linked Hypothesis

eTable 1. Distribution of County-Level Atrazine Use and Gastroschisis by US Census Region

eFigure 2. Overall Rate of Atrazine Use in the US, EPest-High and EPest-Low Methods

eTable 2. Mixed-Effects Logistic Regression Model of Individual- and County-Level Characteristics and Exposures Associated With Gastroschisis, US 2009-2019, Including Births in California and Using EPest-Low

eFigure 3. Mixed-Effects Logistic Regression Model of Individual- and County-Level Characteristics and Exposures Associated With Gastroschisis, US 2009-2019, Including Births in California and Using EPest-Low

eTable 3. Mixed-Effects Logistic Regression Model of Individual- and County-Level Characteristics and Exposures Associated with Gastroschisis, US 2009-2019, Excluding Births in California and Using EPest-High

eFigure 4. Mixed-Effects Logistic Regression Model of Individual- and County-Level Characteristics and Exposures Associated With Gastroschisis, US 2009-2019, Excluding Births in California and Using EPest-High

eTable 4. Mixed-Effects Logistic Regression Model of Individual- and County-Level Characteristics and Exposures Associated With Gastroschisis, US 2009-2019, Excluding Births in California and Using EPest-Low

eFigure 5. Mixed-Effects Logistic Regression Model of Individual- and County-Level Characteristics and Exposures Associated With Gastroschisis, US 2009-2019, Excluding Births in California and Using EPest-Low

This supplemental material has been provided by the authors to give readers additional information about their work.

© 2024 Krishnapura SR et al. *JAMA Network Open.*

eFigure 1. Pathophysiology Model of Gastroschisis Based on the Estrogen-Linked Hypothesis

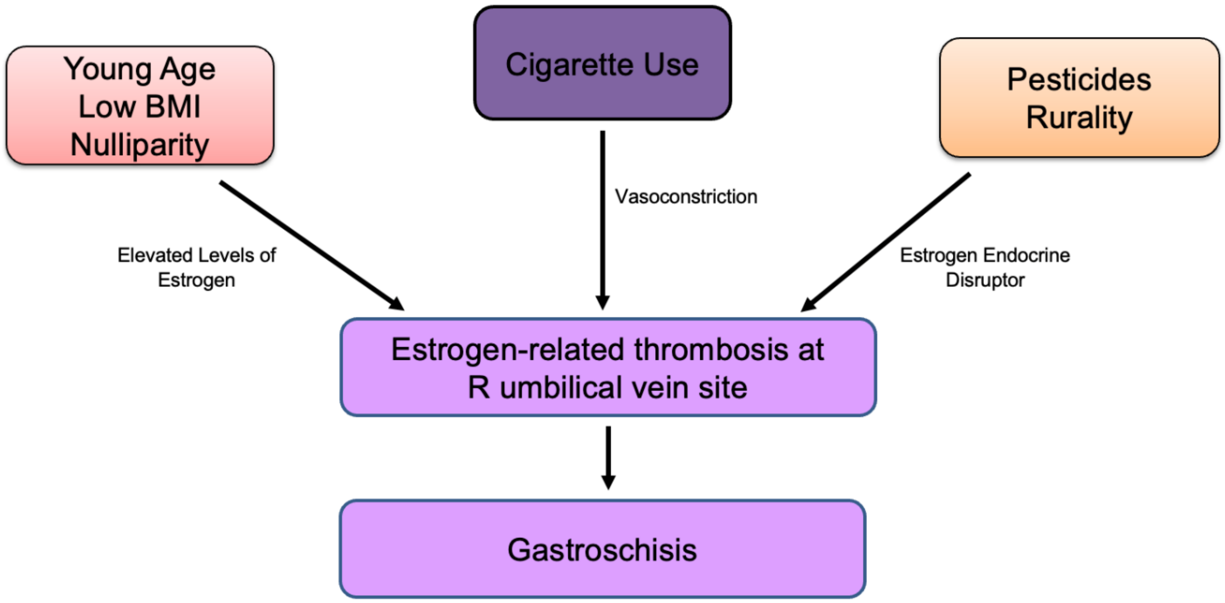

© 2024 Krishnapura SR et al. *JAMA Network Open.*

eTable 1. Distribution of County-Level Atrazine Use and Gastroschisis by US Census Region

YEAR	REGION	BIRTHS	MEAN ATRAZINE USE (KG)	GASTROSCHISIS	GASTROSCHISIS PER 1,000 BIRTHS
**2009**	Midwest	472,330	21,274.41	187	0.40
	Northeast	406,903	4,221.68	104	0.26
	South	1,020,644	4,741.26	312	0.31
	West	878,825	2,647.03	248	0.28
**2010**	Midwest	694,879	20,365.07	284	0.41
	Northeast	403,428	4,082.58	86	0.21
	South	1,104,715	4,852.98	309	0.28
	West	864,984	1,851.92	204	0.24
**2011**	Midwest	808,397	20,955.36	318	0.39
	Northeast	458,892	4,133.20	139	0.30
	South	1,241,602	5,078.46	343	0.28
	West	854,605	2,625.19	218	0.26
**2012**	Midwest	828,041	22,502.77	303	0.37
	Northeast	471,100	4,485.61	109	0.23
	South	1,317,992	5,407.52	371	0.28
	West	857,111	1,782.91	258	0.30
**2013**	Midwest	826,464	23,677.94	272	0.33
	Northeast	471,307	4,906.35	129	0.27
	South	1,388,115	5,784.96	333	0.24
	West	857,175	1,897.23	259	0.30
**2014**	Midwest	836,231	22,590.02	315	0.38
	Northeast	488,045	4,370.82	121	0.25
	South	1,534,146	5,179.69	391	0.25
	West	975,475	1,999.89	299	0.31
**2015**	Midwest	834,616	22,306.18	298	0.36
	Northeast	559,135	3,587.56	161	0.29
	South	1,546,041	5,289.91	353	0.23
	West	963,837	2,157.57	263	0.27
**2016**	Midwest	826,509	23,890.04	269	0.33
	Northeast	623,224	3,551.12	131	0.21
	South	1,531,207	5,892.80	327	0.21
	West	958,144	3,279.20	230	0.24
**2017**	Midwest	808,647	24,845.75	243	0.30
	Northeast	614,160	4,050.33	135	0.22
	South	1,502,324	4,424.62	285	0.19
	West	925,472	4,424.11	262	0.28
**2018**	Midwest	796,671	23,778.48	223	0.28
	Northeast	605,577	4,428.85	124	0.20
	South	1,484,425	4,353.22	272	0.18
	West	898,995	2,975.34	211	0.23
**2019**	Midwest	782,905	23,537.54	253	0.32
	Northeast	596,665	4,360.96	94	0.16
	South	1,477,354	5,126.02	276	0.19
	West	885,252	4,050.52	205	0.23

© 2024 Krishnapura SR et al. *JAMA Network Open.*

eFigure 2. Overall Rate of Atrazine Use in the US, EPest-High and EPest-Low Methods

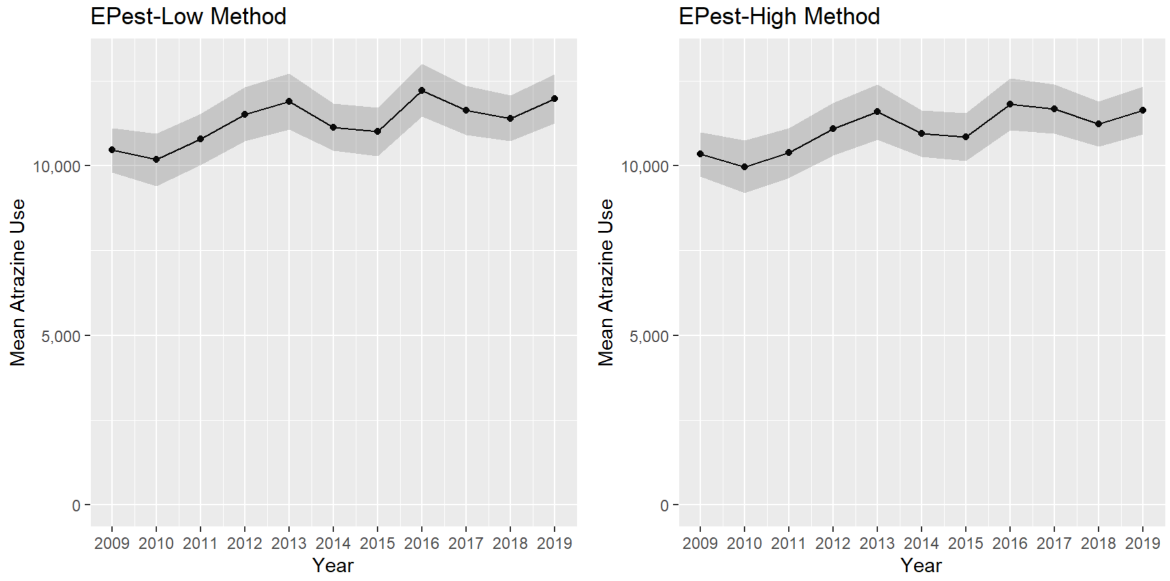

© 2024 Krishnapura SR et al. *JAMA Network Open.*

eTable 2. Mixed-Effects Logistic Regression Model of Individual- and County-Level Characteristics and Exposures Associated With Gastroschisis, US 2009-2019, Including Births in California and Using EPest-Low

	**Model 1: 5-Year Average of Atrazine Use (EPest-low method)** N = 31,892,405		**Model 2: 10-Year Average of Atrazine Use (EPest-low method)** N = 32,939,498		**Model 3: Previous Year Atrazine Use (EPest-low method)** N = 30,137,961	
	aOR (95% C.I.)	P-value	aOR (95% C.I.)	P-value	aOR (95% C.I.)	P-value
**Year**						
2009	1.0 (Reference)		1.0 (Reference)		1.0 (Reference)	
2010	0.96 (0.86 – 1.08)	0.494	0.95 (0.85 – 1.06)	0.360	0.97 (0.86 – 1.08)	0.568
2011	1.08 (0.97 – 1.21)	0.137	1.08 (0.97 – 1.19)	0.170	1.07 (0.96 – 1.19)	0.241
2012	1.11 (1.00 – 1.24)	0.049	1.11 (1.00 – 1.23)	0.043	1.09 (0.98 – 1.21)	0.119
2013	1.06 (0.95 – 1.18)	0.268	1.06 (0.96 – 1.18)	0.246	1.05 (0.95 – 1.18)	0.343
2014	1.16 (1.04 – 1.28)	0.006	1.16 (1.05 – 1.28)	0.004	1.16 (1.04 – 1.29)	0.006
2015	1.15 (1.03 – 1.27)	0.009	1.15 (1.03 – 1.27)	0.009	1.14 (1.03 – 1.27)	0.015
2016	1.05 (0.95 – 1.17)	0.345	1.04 (0.94 – 1.16)	0.411	1.05 (0.94 – 1.17)	0.360
2017	1.09 (0.98 – 1.21)	0.112	1.09 (0.98 – 1.21)	0.125	1.07 (0.96 – 1.19)	0.212
2018	1.02 (0.91 – 1.14)	0.741	1.02 (0.91 – 1.13)	0.754	1.01 (0.90 – 1.13)	0.872
2019	1.09 (0.98 – 1.22)	0.122	1.08 (0.97 – 1.21)	0.139	1.07 (0.96 – 1.20)	0.237
County Characteristics						
**Atrazine Use**						
100,000 kg increase	1.15 (1.02 – 1.30)	0.023	1.21 (1.07 – 1.37)	0.003	1.11 (1.00 – 1.23)	0.053
**Rurality**						
Urban	1.0 (Reference)		1.0 (Reference)		1.0 (Reference)	
Rural Adjacent	1.07 (0.99 – 1.15)	0.079	1.07 (1.00 – 1.16)	0.065	1.07 (0.99 – 1.16)	0.071
Rural Remote	1.12 (1.02 – 1.23)	0.020	1.12 (1.02 – 1.23)	0.013	1.10 (1.00 – 1.21)	0.054
Individual Characteristics						
**Maternal Age**	0.87 (0.86 – 0.87)	<0.001	0.87 (0.86 – 0.87)	<0.001	0.87 (0.86 – 0.87)	<0.001
**Maternal Race and Ethnicity**						
Hispanic	0.89 (0.83 – 0.94)	<0.001	0.89 (0.84 – 0.95)	<0.001	0.89 (0.83 – 0.94)	<0.001
Non-Hispanic Black	0.56 (0.52 – 0.61)	<0.001	0.56 (0.52 – 0.60)	<0.001	0.56 (0.52 – 0.61)	<0.001
Non-Hispanic White	1.0 (Reference)		1.0 (Reference)		1.0 (Reference)	
Other[a]	0.79 (0.71 – 0.88)	<0.001	0.78 (0.70 – 0.87)	<0.001	0.80 (0.72 – 0.90)	<0.001
**Maternal BMI**	0.94 (0.94 – 0.94)	<0.001	0.94 (0.94 – 0.94)	<0.001	0.94 (0.94 – 0.94)	<0.001
**Cigarette Use**	1.68 (1.59 – 1.78)	<0.001	1.69 (1.60 – 1.79)	<0.001	1.69 (1.60 – 1.79)	<0.001
**# of Previous births**	0.91 (0.89 – 0.94)	<0.001	0.91 (0.89 – 0.93)	<0.001	0.91 (0.89 – 0.93)	<0.001
**Chlamydia**	1.15 (1.03 – 1.27)	0.012	1.15 (1.04 – 1.28)	0.008	1.13 (1.02 – 1.26)	0.025
**Payment**						
Medicaid	1.0 (Reference)		1.0 (Reference)		1.0 (Reference)	
Private Insurance	0.73 (0.69 – 0.77)	<0.001	0.73 (0.69 – 0.77)	<0.001	0.73 (0.69 – 0.77)	<0.001
Self-Pay	0.63 (0.55 – 0.72)	<0.001	0.64 (0.56 – 0.73)	<0.001	0.63 (0.55 – 0.72)	<0.001
Other	0.88 (0.80 – 0.98)	0.018	0.89 (0.80 – 0.99)	0.024	0.90 (0.81 – 1.00)	0.058

[a]Non-Hispanic American Indian or Alaskan Native and Asian or Pacific Islander

© 2024 Krishnapura SR et al. *JAMA Network Open.*

eFigure 3. Mixed-Effects Logistic Regression Model of Individual- and County-Level Characteristics and Exposures Associated With Gastroschisis, US 2009-2019, Including Births in California and Using EPest-Low; a) previous year, b) 5-year average, c) 10-year average.

a)

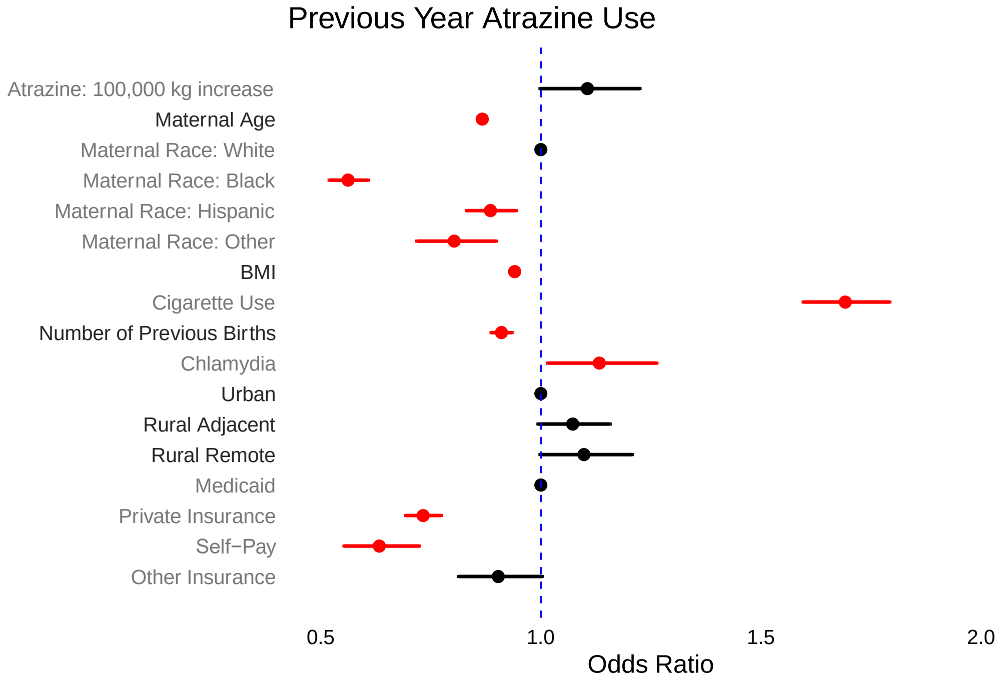

b)

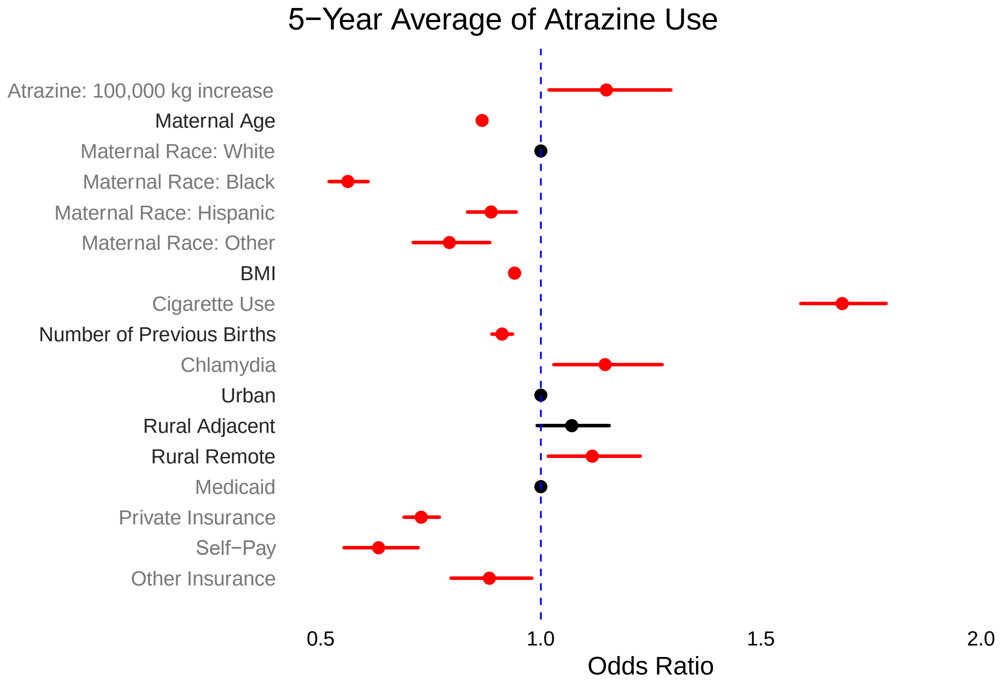

© 2024 Krishnapura SR et al. *JAMA Network Open.*

c)

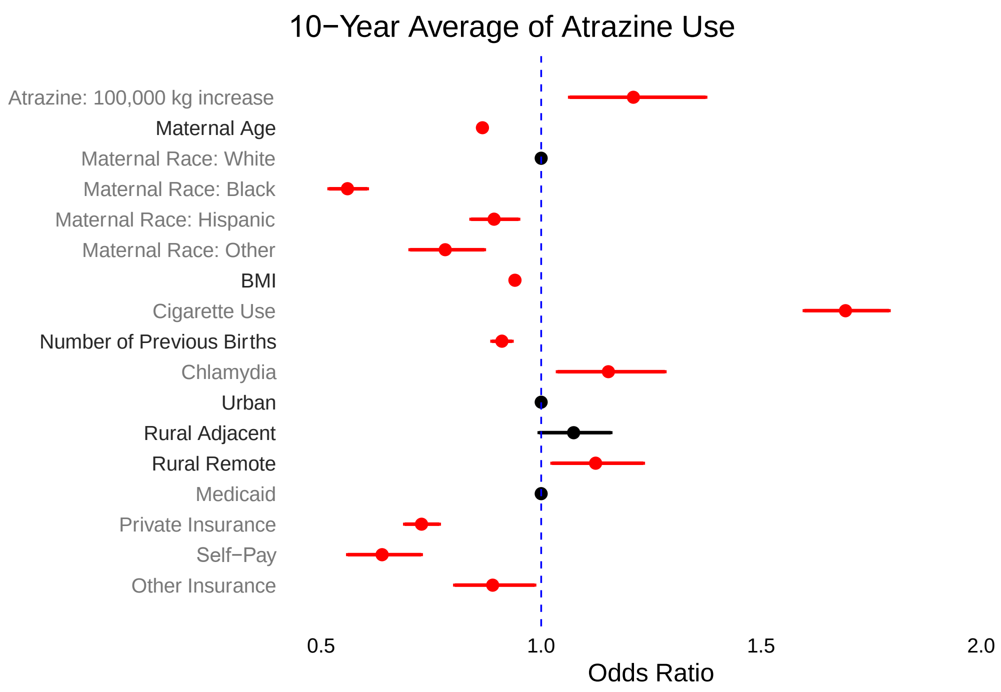

© 2024 Krishnapura SR et al. *JAMA Network Open.*

eTable 3. Mixed-Effects Logistic Regression Model of Individual- and County-Level Characteristics and Exposures Associated With Gastroschisis, US 2009-2019, Excluding Births in California and Using EPest-High

	Model 1: 5-Year Average of Atrazine Use (EPest-high method) N = 29,278,674		Model 2: 10-Year Average of Atrazine Use (EPest-high method) N = 29,484,165		Model 3: Previous Year Atrazine Use (EPest-high method) N = 28,613,331	
	aOR (95% C.I.)	P-value	aOR (95% C.I.)	P-value	aOR (95% C.I.)	P-value
**Year**						
2009	1.0 (Reference)		1.0 (Reference)		1.0 (Reference)	
2010	0.98 (0.87 – 1.09)	0.673	0.97 (0.87 – 1.09)	0.664	0.98 (0.88 – 1.10)	0.768
2011	1.08 (0.97 – 1.20)	0.171	1.08 (0.97 – 1.20)	0.172	1.07 (0.96 – 1.19)	0.238
2012	1.08 (0.97 – 1.20)	0.170	1.08 (0.97 – 1.21)	0.162	1.08 (0.97 – 1.20)	0.171
2013	1.04 (0.93 – 1.16)	0.538	1.04 (0.93 – 1.16)	0.536	1.03 (0.93 – 1.16)	0.555
2014	1.13 (1.02 – 1.26)	0.021	1.13 (1.02 – 1.26)	0.021	1.14 (1.02 – 1.26)	0.019
2015	1.11 (1.00 – 1.23)	0.059	1.11 (1.00 – 1.23)	0.058	1.11 (0.99 – 1.23)	0.063
2016	1.04 (0.93 – 1.16)	0.498	1.04 (0.93 – 1.16)	0.494	1.04 (0.93 – 1.16)	0.501
2017	1.05 (0.94 – 1.17)	0.398	1.05 (0.94 – 1.17)	0.416	1.05 (0.94 – 1.18)	0.349
2018	0.99 (0.89 – 1.11)	0.883	0.99 (0.88 – 1.11)	0.850	0.99 (0.89 – 1.11)	0.914
2019	1.06 (0.94 – 1.18)	0.333	1.06 (0.94 – 1.18)	0.334	1.04 (0.93 – 1.16)	0.525
County Characteristics						
**Atrazine Use**						
100,000 kg increase	1.14 (1.01 – 1.28)	0.029	1.19 (1.05 – 1.35)	0.007	1.11 (1.00 – 1.23)	0.042
**Rurality**						
Urban	Reference		Reference		Reference	
Rural Adjacent	1.06 (0.99 – 1.15)	0.110	1.06 (0.98 – 1.15)	0.117	1.07 (0.99 – 1.15)	0.088
Rural Remote	1.11 (1.01 – 1.21)	0.031	1.11 (1.01 – 1.22)	0.027	1.10 (1.00 – 1.20)	0.048
Individual Characteristics						
**Maternal Age**	0.87 (0.86 – 0.87)	<0.001	0.87 (0.86 – 0.87)	<0.001	0.87 (0.86 – 0.87)	<0.001
**Maternal Race and Ethnicity**						
Hispanic	0.90 (0.84 – 0.96)	0.001	0.90 (0.84 – 0.96)	0.001	0.90 (0.84 – 0.96)	0.001
Non-Hispanic Black	0.57 (0.52 – 0.61)	<0.001	0.57 (0.52 – 0.61)	<0.001	0.57 (0.52 – 0.61)	<0.001
Non-Hispanic White	1.0 (Reference)		1.0 (Reference)		1.0 (Reference)	
Other[a]	0.80 (0.72 – 0.90)	<0.001	0.80 (0.71 – 0.90)	<0.001	0.82 (0.73 – 0.92)	0.001
**Maternal BMI**	0.94 (0.94 – 0.94)	<0.001	0.94 (0.94 – 0.94)	<0.001	0.94 (0.94 – 0.94)	<0.001
**Cigarette Use**	1.68 (1.59 – 1.78)	<0.001	1.69 (1.59 – 1.79)	<0.001	1.69 (1.59 – 1.79)	<0.001
**# of Previous births**	0.91 (0.89 – 0.94)	<0.001	0.91 (0.89 – 0.94)	<0.001	0.91 (0.89 – 0.94)	<0.001
**Chlamydia**	1.14 (1.02 – 1.27)	0.019	1.14 (1.03 – 1.27)	0.015	1.14 (1.02 – 1.27)	0.022
**Payment**						
Medicaid	1.0 (Reference)		1.0 (Reference)		1.0 (Reference)	
Private Insurance	0.73 (0.69 – 0.77)	<0.001	0.73 (0.69 – 0.77)	<0.001	0.73 (0.69 – 0.77)	<0.001
Self-Pay	0.64 (0.56 – 0.73)	<0.001	0.64 (0.56 – 0.73)	<0.001	0.65 (0.57 – 0.74)	<0.001
Other	0.91 (0.82 – 1.00)	0.062	0.91 (0.82 – 1.01)	0.062	0.91 (0.82 – 1.01)	0.066

[a]Non-Hispanic American Indian or Alaskan Native and Asian or Pacific Islander

© 2024 Krishnapura SR et al. *JAMA Network Open.*

eFigure 4. Mixed-Effects Logistic Regression Model of Individual- and County-Level Characteristics and Exposures Associated With Gastroschisis, US 2009-2019, Excluding Births in California and Using EPest-High; a) previous year, b) 5-year average, c) 10-year average.

a)

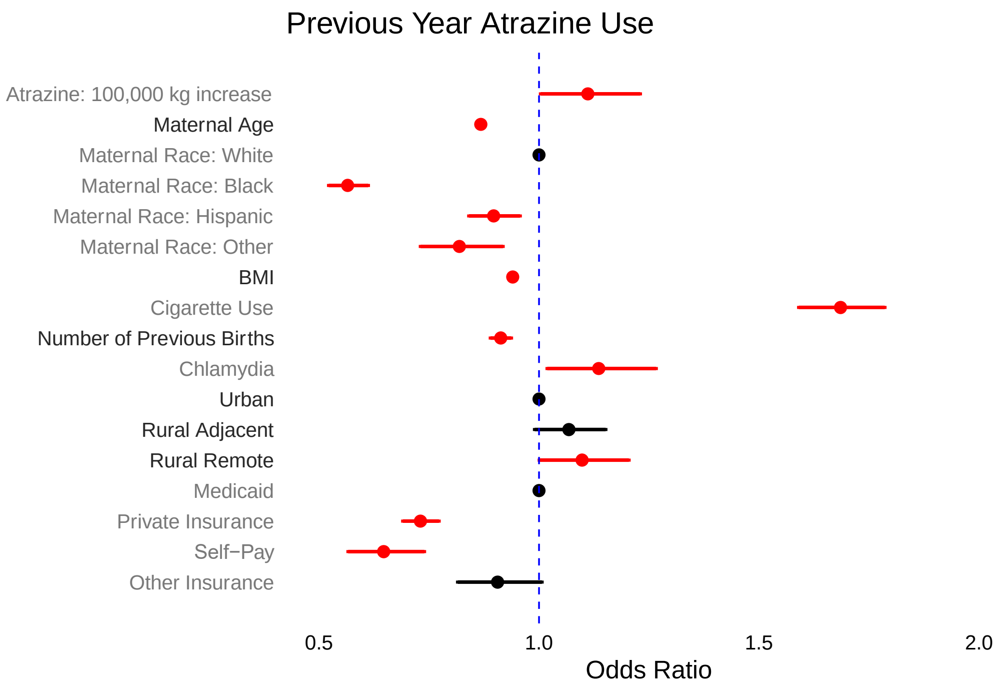

b)

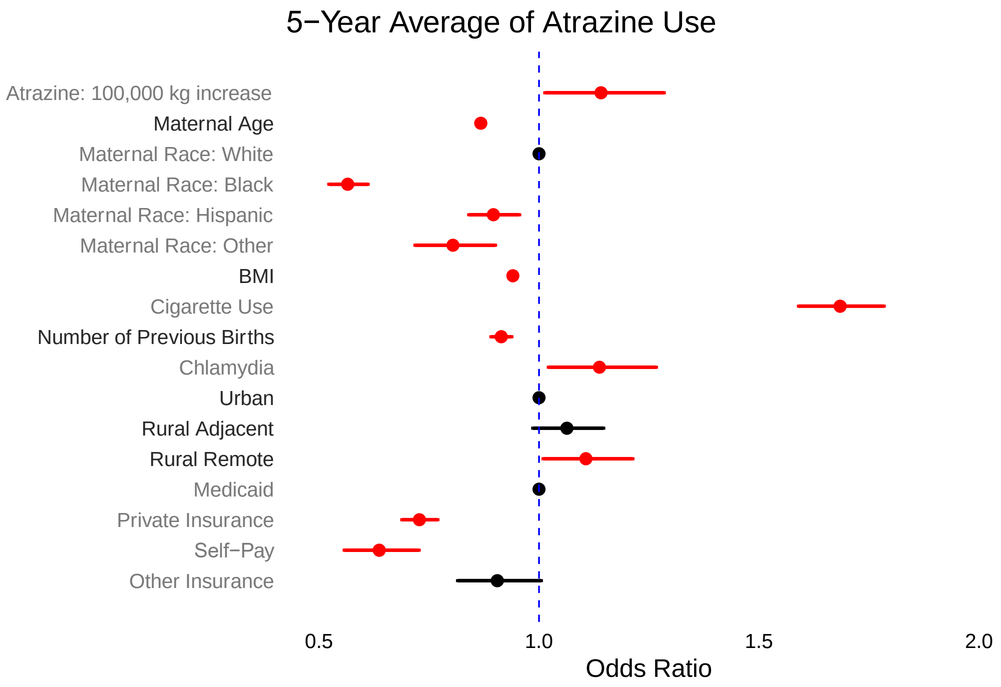

© 2024 Krishnapura SR et al. *JAMA Network Open.*

c)

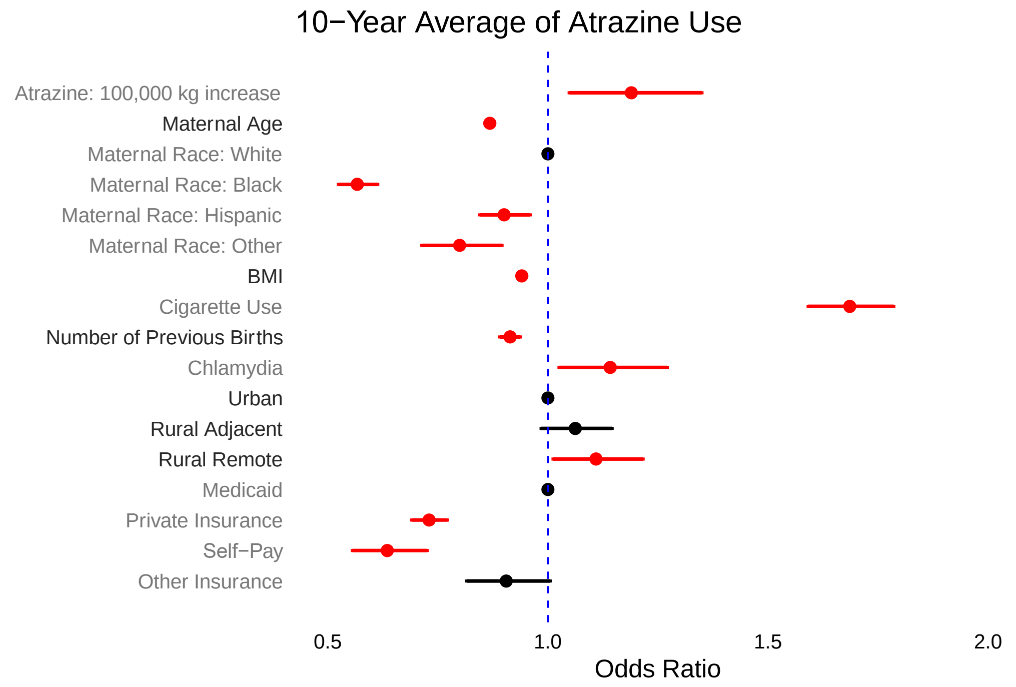

© 2024 Krishnapura SR et al. *JAMA Network Open.*

eTable 4. Mixed-Effects Logistic Regression Model of Individual- and County-Level Characteristics and Exposures Associated With Gastroschisis, US 2009-2019, Excluding Births in California and Using EPest-Low

	**Model 1: 5-Year Average of Atrazine Use (EPest-low method)** N = 29,018,748		**Model 2: 10-Year Average of Atrazine Use (EPest-low method)** N = 29,302,112		**Model 3: Previous Year Atrazine Use (EPest-low method)** N = 28,011,081	
	aOR (95% C.I.)	P-value	aOR (95% C.I.)	P-value	aOR (95% C.I.)	P-value
**Year**						
2009	1.0 (Reference)		1.0 (Reference)		1.0 (Reference)	
2010	0.98 (0.87 – 1.10)	0.718	0.98 (0.87 – 1.10)	0.707	0.98 (0.87 – 1.10)	0.705
2011	1.08 (0.97 – 1.21)	0.163	1.08 (0.97 – 1.20)	0.175	1.07 (0.95 – 1.19)	0.265
2012	1.09 (0.97 – 1.21)	0.139	1.08 (0.97 – 1.21)	0.149	1.07 (0.96 – 1.20)	0.212
2013	1.04 (0.93 – 1.16)	0.476	1.04 (0.93 – 1.16)	0.476	1.04 (0.93 – 1.16)	0.530
2014	1.14 (1.03 – 1.27)	0.015	1.14 (1.02 – 1.27)	0.017	1.15 (1.03 – 1.28)	0.011
2015	1.12 (1.00 – 1.24)	0.044	1.12 (1.00 – 1.24)	0.046	1.12 (1.00 – 1.25)	0.044
2016	1.04 (0.93 – 1.16)	0.469	1.04 (0.93 – 1.16)	0.487	1.04 (0.93 – 1.16)	0.486
2017	1.05 (0.94 – 1.18)	0.350	1.05 (0.94 – 1.17)	0.380	1.05 (0.94 – 1.17)	0.424
2018	0.99 (0.89 – 1.11)	0.919	0.99 (0.89 – 1.11)	0.890	0.99 (0.89 – 1.11)	0.913
2019	1.06 (0.95 – 1.19)	0.277	1.06 (0.95 – 1.19)	0.311	1.04 (0.92 – 1.16)	0.539
County Characteristics						
**Atrazine Use**						
100,000 kg increase	1.14 (1.01 – 1.28)	0.036	1.19 (1.05 – 1.35)	0.008	1.10 (1.00 – 1.22)	0.063
**Rurality**						
Urban	1.0 (Reference)		1.0 (Reference)		1.0 (Reference)	
Rural Adjacent	1.07 (0.99 – 1.15)	0.101	1.07 (0.99 – 1.15)	0.101	1.07 (0.99 – 1.16)	0.078
Rural Remote	1.11 (1.01 – 1.21)	0.032	1.11 (1.02 – 1.22)	0.023	1.09 (1.00 – 1.20)	0.061
Individual Characteristics						
**Maternal Age**	0.87 (0.86 – 0.87)	<0.001	0.87 (0.86 – 0.87)	<0.001	0.87 (0.86 – 0.87)	<0.001
**Maternal Race and Ethnicity**						
Hispanic	0.90 (0.84 – 0.96)	0.001	0.90 (0.84 – 0.96)	0.001	0.89 (0.84 – 0.95)	0.001
Non-Hispanic Black	0.56 (0.52 – 0.61)	<0.001	0.56 (0.52 – 0.61)	<0.001	0.57 (0.52 – 0.61)	<0.001
Non-Hispanic White	1.0 (Reference)		1.0 (Reference)		1.0 (Reference)	
Other[a]	0.80 (0.72 – 0.90)	<0.001	0.80 (0.72 – 0.90)	<0.001	0.80 (0.71 – 0.90)	<0.001
**Maternal BMI**	0.94 (0.94 – 0.94)	<0.001	0.94 (0.94 – 0.94)	<0.001	0.94 (0.94 – 0.94)	<0.001
**Cigarette Use**	1.68 (1.58 – 1.78)	<0.001	1.68 (1.59 – 1.78)	<0.001	1.68 (1.59 – 1.78)	<0.001
**# of Previous births**	0.91 (0.89 – 0.94)	<0.001	0.91 (0.89 – 0.94)	<0.001	0.91 (0.89 – 0.94)	<0.001
**Chlamydia**	1.13 (1.02 – 1.26)	0.023	1.15 (1.03 – 1.28)	0.013	1.12 (1.01 – 1.25)	0.038
**Payment**						
Medicaid	1.0 (Reference)		1.0 (Reference)		1.0 (Reference)	
Private Insurance	0.73 (0.69 – 0.77)	<0.001	0.73 (0.69 – 0.77)	<0.001	0.73 (0.69 – 0.78)	<0.001
Self-Pay	0.64 (0.56 – 0.73)	<0.001	0.64 (0.56 – 0.73)	<0.001	0.65 (0.57 – 0.74)	<0.001
Other	0.90 (0.81 – 1.00)	0.053	0.90 (0.81 – 1.00)	0.057	0.92 (0.82 – 1.02)	0.112

[a]Non-Hispanic American Indian or Alaskan Native and Asian or Pacific Islander

© 2024 Krishnapura SR et al. *JAMA Network Open.*

eFigure 5. Mixed-Effects Logistic Regression Model of Individual- and County-Level Characteristics and Exposures Associated With Gastroschisis, US 2009-2019, Excluding Births in California and Using EPest-Low; a) previous year, b) 5-year average, c) 10-year average.

a)

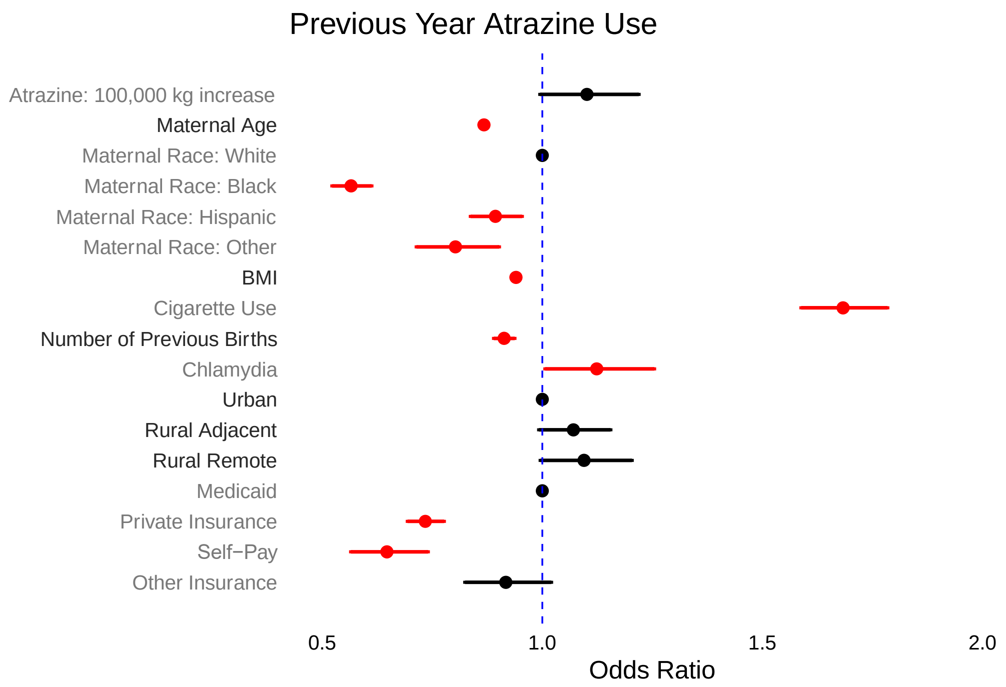

b)

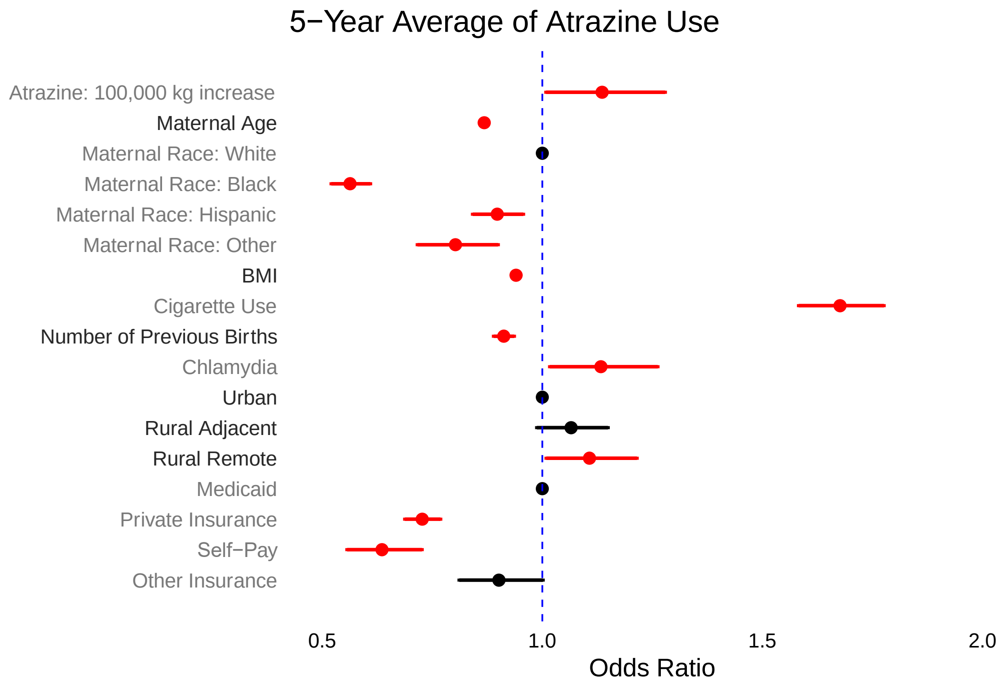

© 2024 Krishnapura SR et al. *JAMA Network Open.*

c)

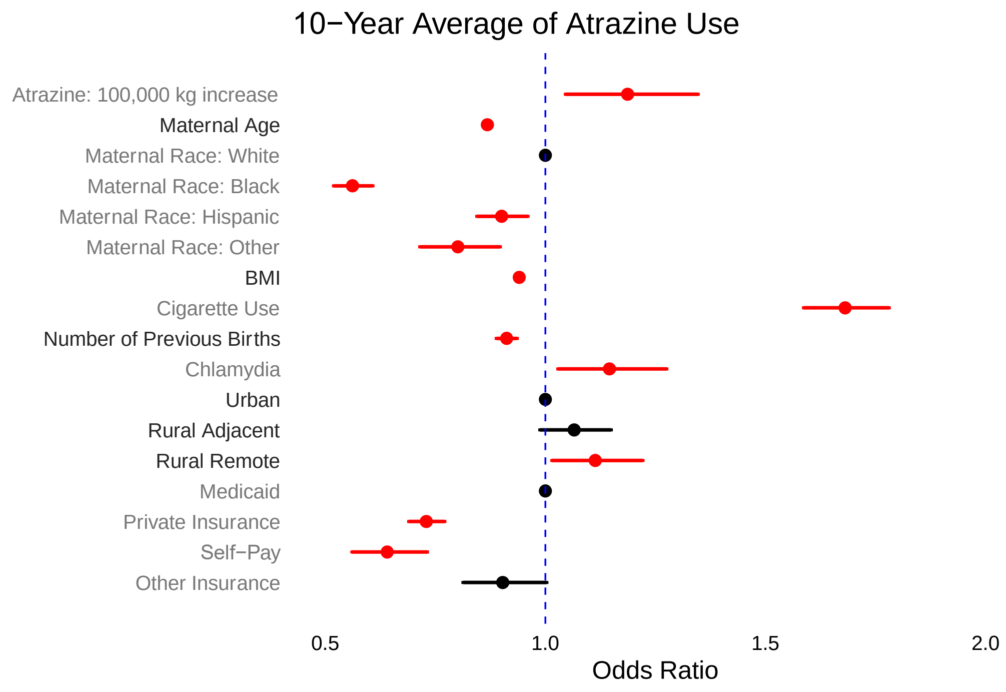

© 2024 Krishnapura SR et al. *JAMA Network Open.*
