# Supplementary material for: County-Level Atrazine Use and Gastroschisis
Source: JAMA Netw Open. 2024 May 6;7(5):e2410056. doi: 10.1001/jamanetworkopen.2024.10056 (PMC11074809; doi:10.1001/jamanetworkopen.2024.10056)
Supplement: Supplement 2. — Data Sharing Statement [file jamanetwopen-e2410056-s002.pdf]

Data Sharing Statement

Krishnapura. County-Level Atrazine Use and Gastroschisis. *JAMA Netw Open*. Published May 06, 2024. doi:10.1001/jamanetworkopen.2024.10056

Data
Data available: No

Additional Information
Explanation for why data not available: Data are publicly available.
